# Genetic Association Analysis of Anti-VEGF Treatment Response in Neovascular Age-Related Macular Degeneration

**DOI:** 10.3390/ijms23116094

**Published:** 2022-05-29

**Authors:** Tobias Strunz, Michael Pöllmann, Maria-Andreea Gamulescu, Svenja Tamm, Bernhard H. F. Weber

**Affiliations:** 1 Institute of Human Genetics, University of Regensburg, 93053 Regensburg, Germany; tobias.strunz@klinik.uni-regensburg.de; 2 Department of Ophthalmology, Regensburg University Medical Center, 93053 Regensburg, Germany; michael.poellmann@klinik.uni-regensburg.de (M.P.); andreea.gamulescu@klinik.uni-regensburg.de (M.-A.G.); 3 Institute of Clinical Human Genetics, University Hospital Regensburg, 93053 Regensburg, Germany

**Keywords:** age-related macular degeneration, AMD, anti-VEGF treatment, observational study, genetic analysis

## Abstract

Anti-VEGF treatment for neovascular age-related macular degeneration (nAMD) has been FDA-approved in 2004, and since then has helped tens of thousands of patients worldwide to preserve vision. Still, treatment responses vary widely, emphasizing the need for genetic biomarkers to robustly separate responders from non-responders. Here, we report the findings of an observational study compromising 179 treatment-naïve nAMD patients and their reaction to treatment after three monthly doses of anti-VEGF antibodies. We show that established criteria of treatment response such as visual acuity and central retinal thickness successfully divides our cohort into 128 responders and 51 non-responders. Nevertheless, retinal thickness around the fovea revealed significant reaction to treatment even in the formally categorized non-responders. To elucidate genetic effects underlying our criteria, we conducted an undirected genome-wide association study followed by a directed replication study of 30 previously reported genetic variants. Remarkably, both approaches failed to result in significant findings, suggesting study-specific effects were confounding the present and previous discovery studies. Of note, all studies so far are greatly underpowered, hampering interpretation of genetic findings. In consequence, we highlight the need for an extensive phenotyping study with sample sizes exceeding at least 15,000 to reliably assess anti-VEGF treatment responses in nAMD.

## 1. Introduction

Age-related macular degeneration (AMD) is a multifactorial disease with late-onset affecting central vision of millions of people worldwide [1]. Disease risk is known to be conferred by environmental factors, such as smoking or ageing, as well as by genetic predisposition [2,3]. In its early stages, AMD often remains unnoticed by the patient, while progression to late-stage forms can lead to gradual or rapid vision loss. The clinical manifestation of late-stage AMD is characterized by the degradation of retinal pigment epithelium (RPE) and photoreceptor cells, and can be further delineated according to the presence (neovascular AMD, nAMD) or absence (geographic atrophy, GA) of newly formed blood vessels in the disease process [4].

To date, several treatment options are available for nAMD [5,6,7], all based on a unifying principle that aims at inhibiting angiogenesis by applying intravitreal VEGF neutralization. Successful therapy, as observed for example by anti-VEGF antibody treatment, blocks immature vessel formation and bleeding, resulting in the reduction of intraretinal (IRF) and subretinal (SRF) fluid accumulation. Multiple treatment regimens for antibody applications are used, ranging from monthly doses to a pro re nata (PRN) or a treat and extend (TAE) strategy [6,8]. Independent of the specific procedure, most of the patients report a reduction of symptoms even after the first intravitreal injection. Several options have emerged to determine treatment success, based on either optical coherence tomography (OCT) or clinical parameters, such as visual acuity (VA). So far, no gold-standard has been defined, although anti-VEGF treatment for nAMD has been available for over ten years.

A number of clinical and observational studies consistently report that retinal fluid is sometimes only partially dissolved in individuals after treatment [9,10]. Further, up to 10% of treated patients show even a worsening of symptoms [6,11,12]. The observation of “non-responders” frequently raises the question of whether genetic variants could influence the treatment response and consequently may serve as biomarkers to distinguish or to predict outcome [13]. Several studies have been performed reporting significant association of multiple genetic markers, while it became clear in subsequent meta-analyses and reviews that most results are contradictory or appear highly study dependent. This results in a scenario where it is unclear how to use the genetic association data for decision making when it comes to patient management and concepts in improvement of treatment outcome [14,15,16].

Here, we report the findings of an observational study combined with a critical review of the current literature. This provides further insight into the present state of knowledge, specifically with regard to reproducibility of genetic effects underlying nAMD treatment response. On this basis, we discuss some of the reasons for study-specific effects and propose a future study design required to precisely and more comprehensively phenotype nAMD treatment response.

## 2. Materials and Methods

### 2.1. Observational Study Design

We performed an observational study including a total of 183 consecutive nAMD patients visiting our specialized outpatient clinic at the University Eye Clinic Regensburg between July 2018 and January 2021. Inclusion criteria for all study patients were defined as neovascularization secondary to AMD and age greater than 50 years. Exclusion criteria included any retinal disease other than nAMD which could be responsible for the presence of fluid. In addition, patients were excluded if they previously underwent retinal surgery or showed any signs of intraocular inflammation. All probands were treatment-naïve for nAMD. After an initial clinical examination (baseline measurement), patients received three monthly injections with either Eylea (Bayer AG, Leverkusen, Germany) or Lucentis (Novartis, Basel, Switzerland). Follow-up was performed to determine treatment response one month after the third injection (treatment measurement). The study and the data collection strictly adhered to the declaration of Helsinki principles. Ethical approval was given by the local Institutional Review Board (Reference-ID 18-936-101, dated 15.03.2018, University of Regensburg). The data collected included demographic variables, OCT measurements, lens characteristics, VA, and comorbidities like hypertension or diabetes.

### 2.2. Clinical Data Acquisition

Prior to the acquisition of clinical data, the diagnosis of nAMD was confirmed by fundus fluorescein angiography. OCT images were generated with an HRA + OCT Spectralis device (Heidelberg Engineering, Heidelberg, Germany). For all patients, a radial scan with six-line scans was acquired, focused on the fovea centralis at baseline and after treatment. Evaluation of OCT scans was conducted by the Heidelberg Eye Explorer (HRA Viewer version 6.16.7.0) (Heidelberg Engineering, Heidelberg, Germany). First, central retinal thickness (CRT) was determined by covering the distance from Bruch’s membrane to the internal limiting membrane at the fovea centralis. CRT was manually measured in the horizontal or the vertical OCT scan, selecting the image on which the fovea was best recognizable. We next examined all line scans to identify the angle showing the thickest retinal thickness (TRT), which was determined similar to the CRT (Figure S1). 

Visual acuity was assessed with standard numeric optotypes at 5 m using a Rodavist 300 (Rodenstock, Munich, Germany) or a M 3000 (Möller-Wedel, Wedel, Germany) chart projector. Decimal visual acuities were then converted to logarithm of the minimum angle of resolution (logMAR) scores for statistical analysis.

### 2.3. Genetic Data Acquisition

Genomic DNA was isolated from saliva samples applying the Oragene-DNA OG-500 Kit (DNA Genotek, Ottawa, Ontario, Canada). DNA samples were genotyped on an Axiom Precision Medicine Research Array (Affymetrix, Santa Clara, CA, USA). Genotype calling was performed with the help of the Axiom analysis suite desktop application (v5.0.1) applying the “Best Practices” workflow. This led to the exclusion of three samples not fulfilling the manufacturers quality requirements, leaving 180 individuals for further analysis. A total of 861,305 genetic variants were extracted in the variant call format (VCF) [17]. Quality control (QC) on the variant level considered filtering for autosomal variants, removal of monomorphic variants and of variants with a deviation from Hardy–Weinberg equilibrium (HWE) at *p*-value < 1 × 10^−6^ and a call rate < 95%. To determine ethnicity of samples, a principal component analysis (PCA) was carried out in R (Version 3.3.1) [18] using the *snpgdsPCA* [19] function based on 100,000 random genetic variants of each sample and the corresponding genotype information of the 1000 Genomes Project reference panel (Phase 3, release 20130502) [20]. The first two principal components were plotted to determine ethnicity (Figure S2). Only samples clustering next to the European reference individuals were included to consider the known variation of haplotype structures between populations. This criterion excluded one sample. Next, genotypes of the remaining 179 samples were phased with *SHAPEIT2* (Version 2.r904) [21], and then imputed to the 1000 Genomes Project Phase 3 reference panel [20] by IMPUTE2 (Version 2.3.2) [22]. For post-imputation QC, the output files were converted to VCF containing confidently imputed variants (quality threshold > 0.3) in an estimated allele dosage format. Finally, a minor allele frequency filter of 5% and an additional HWE test (*p*-value < 1 × 10^−6^) were applied, resulting in 6,908,005 genetic variants available for analysis. Handling of VCF data was performed by VCFtools (Version 0.1.17) [17].

### 2.4. Definition of Treatment Response

We evaluated OCT images of all 179 study participants with available clinical and genetic data regarding the presence of retinal fluid before and after treatment. This identified 107 of 179 study participants revealing no signs of IRF or SRF after completion of the treatment regimen. These 107 individuals were assigned to the responder group. We further chose two conservative criteria to identify probands with a positive response to treatment, and selected for individuals whose CRT decreased strongly (> 200 µm) or whose VA increased for at least 10 letters (gain of at least 0.2 logMAR). These criteria resulted in 128 responders and 51 non-responders.

### 2.5. Genetic Association Testing and Statistic Analysis

Association analysis of genotype and phenotype data was performed with the help of the logistic regression model (*--glm* function) provided by PLINK 2.0 (version v2.00a3LM) [23]. Covariates included baseline CRT, baseline VA, age, gender, treatment type, eye position, and the first three principal components from the genotype PCA. Comparison of physiological and clinical parameters between study groups was conducted using a pairwise Wilcoxon test, adjusted for multiple testing based on the false discovery rate (FDR) approach [24]. The R libraries ggplot2 [25] and qqman [26] were applied to visualize results.

### 2.6. Literature Search

A systematic literature search was performed to comprehensively identify studies from January 2014 to December 2021, investigating genetic effects underlying anti-VEGF nAMD treatment response. We inquired PubMed [27] by querying the combined search term “(((AMD) OR (age related macular degeneration)) AND (((ranibizumab)) OR (bevacizumab) OR (brolucizumab) OR (aflibercept) OR (treatment)) AND ((genetic*) OR (polymorphism) OR (pharmacogenetic)))”. This identified an initial 1720 publications which were manually filtered by abstract and full text assessment, leaving a final of 53 studies of interest (Table S1). We further added an extensive review by Fauser and Lambrou (2015) [14], comprehensively covering genetic studies in nAMD treatment response for the period before 2014.

### 2.7. Quality Control Measures

Significance thresholds and definitions for treatment responses varied widely in the 54 publications selected. To account for this, we extracted all genetic variants that were considered significant in the respective study, usually in a range of *p*-values below 0.05 or, alternatively, with replication data from an independent cohort. Fauser and Lambrou (2015) [14] reported an overall total of seven variants, which showed ambiguous but significant results in studies published before 2014. Of note, the reports did not allow to identify unambiguous effect alleles for the respective variants as the individual studies reported conflicting, although significant, effect directions.

We applied LDlink [28] to remove genetic variants in high LD (R^2^ > 0.8). Only the variant, which showed the lowest *p*-value in the respective discovery study (sorted by publication date) was further considered. Additionally, if the orientation of alleles was conflicting with the 1000 Genomes Project reference panel (Phase 3, release 20130502), strand orientation was switched [29]. Rare variant rs55667289 (MAF < 0.01 in Europeans) and variant rs1599988, located in the mitochondrial genome, were removed from the analysis. After QC, a total of 30 variants remained in the analysis and were covered in our genotyping approach (Table S2).

### 2.8. Statistical Power Analysis

Power analysis was conducted in R applying the package *genpwr* [30] based on varying parameters as mentioned in the respective results section.

## 3. Results

### 3.1. Observational Study—Clincial Evaluation

In our observational study we included 179 treatment-naïve nAMD patients (Table 1), who were on average 77.3 years (standard deviation (SD): 7.15) old. Clinical data were collected before (baseline) and one month after three monthly doses of either Eylea or Lucentis (treated), whereas genetic data were obtained at baseline or at any follow-up visit.

**Figure 1:**
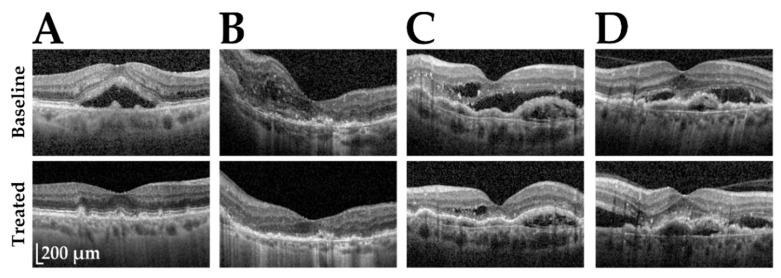
Exemplary optical coherence tomography (OCT) images of nAMD patients and their categorization according to treatment responses. Shown are the OCT images of four patients (**A**–**D**) before (baseline) and after treatment (treated). Individuals in (**A**,**B**) were assigned to the responder group, while probands in (**C**,**D**) were more complex in their response to treatment. The individual in (**C**) was categorized as a responder due to morphological improvements (101 µm decrease in central retinal thickness) and a strong improvement of visual acuity (gain of 0.3 logMAR), although a dry retina was not achieved during the treatment period. The patient in (**D**) was not responsive to the treatment and was assigned as a non-responder.

Based on OCT evaluation, we identified 107 of 179 study participants revealing no signs of IRF or SRF after completion of the treatment regimen (Figure 1A,B). For the remaining individuals, retinal fluid was either partially reduced (Figure 1C) or even increased after treatment (Figure 1D). To unambiguously identify probands with positive response to treatment, we selected for individuals whose CRT decreased strongly (> 200 µm) or whose VA increased for at least 10 letters (gain of at least 0.2 logMAR). These criteria assigned an additional 21 probands with partial reduction of retinal fluid (Figure 1C) to the responder group and left 51 non-responders.

**Figure 2:**
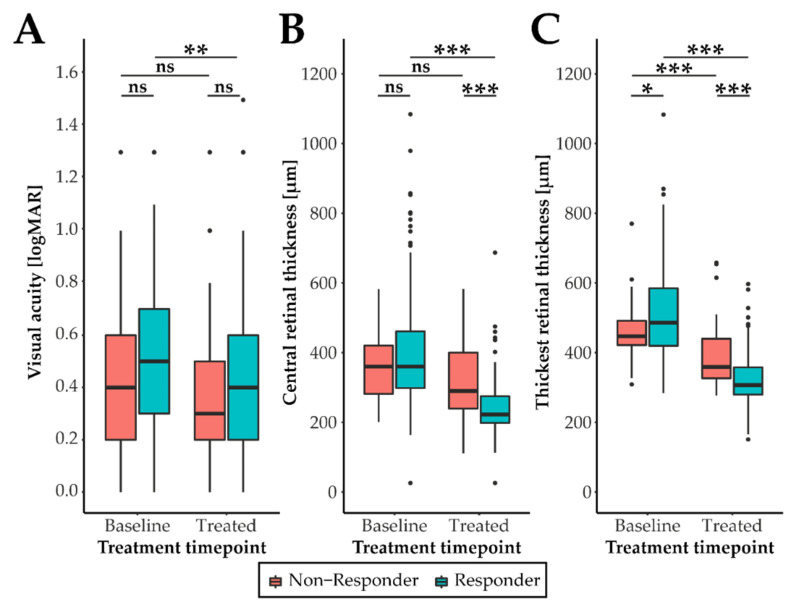
Clinical characteristics of non-responders and responders. The 179 study participants were grouped one month after three monthly anti-VEGF nAMD treatments in non-responders or responders according to the presence of retinal fluid or the strong improvement of clinical markers like central retinal thickness (decrease of > 200µm) or visual acuity (gain of >10 letters, logMAR < −0.2). This resulted in 128 responders and 51 non-responders. Shown are the measurements of visual acuity (**A**), central retinal thickness (**B**) and thickest retinal thickness (**C**) at baseline and after treatment. A pairwise Wilcoxon test, adjusted for multiple testing, was performed to determine significant differences between the two groups. Adjusted *p*-value thresholds: ns ≥0.05, * <0.05, ** <0.01, and *** <0.001. In (**A**), a single outlier of the responder group with visual acuity of 2.2 logMAR at baseline is not depicted to facilitate optimal scaling of the data.

VA at baseline did not differ between responders and non-responders (adjusted *p*-value 0.11), but improved significantly after treatment in the responder group (adjusted *p*-value 0.005) (Figure 2A). We observed no significant change in VA in the non-responders comparing baseline to treated individuals (adjusted *p*-value 0.58). Prominent changes were observed with OCT monitoring. Mean CRT in the responder group decreased significantly from 411.2 µm (SD 175.4) to 243.74 µm (SD 80.02) (adjusted *p*-value 2.4 × 10^−22^) (Figure 2B, Table 1). No significant changes were detected in the non-responder group at baseline versus treated (adjusted *p*-value 0.056).

Evaluations of the patients’ OCT scans showed some lesions to be exclusively extrafoveal (Figure 1B). This was followed up as a novel parameter by measuring the overall thickest part of the retina (TRT) before and after treatment (Figure 2C). A significant reduction of TRT was detected in both the responders (adjusted *p*-value 3.7 × 10^−31^) and the non-responders (adjusted *p*-value 3.0 × 10^−07^). Although the reduction was stronger in the responder group, the findings suggest that the non-responder group still contains individuals responding to the treatment. An example of a case which was grouped as a non-responder is provided in Figure S1. Although revealing a clinical treatment response in TRT, there is still residual intraretinal fluid which resulted in the failure of the CRT and VA criteria. Nevertheless, a further subgrouping of our cohort by including TRT was not feasible due to a greatly reduced sample size of the non-responders unsuited for subsequent genetic analysis. Of note, the mean TRT at baseline was 55.01 µm higher in the responder group compared to the non-responders (adjusted *p*-value 0.028).

### 3.2. Genome-Wide Association Study of Treatment Response

**Figure 3:**
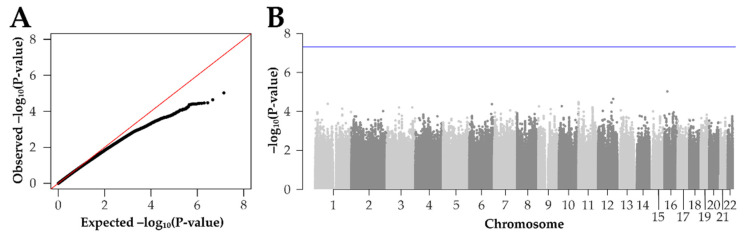
Genome-wide association study of anti-VEGF nAMD treatment response. We performed a logistic regression model to test the association of genetic variants with anti-VEGF nAMD treatment response. The study included 128 responders and 51 non-responders. (**A**) A quantile–quantile (QQ) plot of the results compares observed *p*-values with those expected under the null hypothesis of no association. (**B**) The Manhattan plot shows the -log_10_ *p*-values and positions of 6,908,005 variants. The blue line indicates the threshold for genome-wide significance at 5 × 10^−8^.

We then analyzed treatment response in the responder and non-responder group for contribution of genetic factors. First, we applied an unbiased approach with imputed genotypes encompassing a total of 6,908,005 genetic variants evenly distributed across the autosomes. As covariates, we included baseline CRT, baseline VA, age, gender, treatment type, eye position, and the first three genotype principal components in the analysis. Our logistic regression model resulted in the lowest *p*-value of 9.3 × 10^−06^ for rs35058660 at 16:19243209, which fails to reach the threshold for genome-wide significance (*p*-value < 5 × 10^−08^) (Figure 3). A quantile–quantile (QQ) plot of the GWAS results demonstrated a deflation of observed *p*-values, implying that our sample size for a genome-wide analysis of treatment response is not sufficient (Figure 3A). Still, summary statistics of all variants are accessible online in the Zenodo database (https://zenodo.org/, accessed on 23 May 2022) to facilitate future meta-analyses.

### 3.3. Targeted Genetic Analysis

Next, we focused on genetic variants and regions with previous reference to association. A comprehensive literature search systematically identified 41 original articles reporting genetic findings in nAMD treatment response (Table S1). In addition, we included five GWAS studies and another seven publications reporting meta-analysis data.

The 48 targeted approaches (without GWAS) investigated on average 16.3 (SD 53.6) genetic variants in 4.6 (SD 12.8) different genomic loci. Fourteen studies found no associations, while half of the 48 studies reported a single significant treatment-associated variant. Another 10 studies reported at least two associated genetic variants. Of the five GWAS, one reported no significant association, three found a single treatment associated variant, and one study identified 3 associated genetic variants. Of note, none of these variants reached genome-wide significance.

To generate a comprehensive list of potential nAMD treatment-associated variants, we additionally included data from Fauser and Lambrou (2015) [14], a review covering all association data prior to 2014. This generated a list of 32 genetic variants (Table S2). Thirty of these were tested in our observational study as they were located on autosomes and fulfilled the minor allele frequency threshold of five percent (Table 2).

Remarkably, none of the thirty variants analyzed reached an adjusted *p*-value threshold of 0.05 in our multivariate logistic regression model (Table 2). To assess whether this effect could be due to missing statistical power in the discovery studies or our own replication cohort, we performed a power analysis under varying assumptions considering effect sizes (odds ratio, OR), significance levels (alpha), and the MAFs of underlying genetic effects (Figure S3). The alpha levels reflect three scenarios including single variant testing (alpha 0.05), multiple variant testing adjusted for multiple testing (alpha 0.001), and genome-wide testing (alpha 5 × 10^−08^). Power analysis reveals that testing of more than one genetic variant in a sample size of 179 will detect a true positive finding only if the OR reaches at least five, given a minor allele frequency above 0.25. Further, genome-wide association studies would require approximately 15,000 samples to identify effects with an OR = 1.5 and a MAF of 0.05 (Figure S3, red).

## 4. Discussion

In the present study, we aimed to identify and/or validate genetic variants associated with anti-VEGF treatment response in an nAMD patient cohort. We included 179 treatment-naïve nAMD probands of which 107 (59.8%) reached a dry retina after the specified treatment regimen of three consecutive anti-VEGF injections within a three-month period. Another 21 (11.8%) probands were also assigned to the responder group due to a drastically improved VA or a greatly reduced CRT. A comparison with the remaining 51 (28.5%) non-responders revealed a significant increase in VA and a significant decrease of CRT in the responder group. However, genetic analysis of response status failed to result in significant findings for both an undirected GWAS approach and a directed analysis of 30 previously reported genetic variants.

Five GWAS have been conducted in the context of nAMD treatment [31,32,33,34,35], ranging in sample size from 295 [35] to 2058 [34], all based on a discovery and a replication cohort. None of the studies identified a variant with genome-wide significance. Remarkably, *p*-values in the replication cohorts consistently were less significant when compared to the respective discovery studies, although the replication cohort mostly included more samples than the discovery study. For example, Lorés-Motta et al. (2018) collected patient samples from several clinics and identified rs12138564 (1: 156,291,600) as a potential marker associated with anti-VEGF nAMD treatment response in a discovery phase including 679 individuals (*p*-value 5.7 × 10^−07^). Validation was done in a multi-center replication cohort of 1380 individuals and resulted in a *p*-value of 0.029 [34]. This may suggest study-specific confounder effects, possibly superimposing true biological and genetic effects.

The available meta-analysis data support a similar conclusion as the reported effects are often small or not reproducible. Of note is a study by Wang et al. (2021) that re-calculates the results of 33 publications on genetic effects of response to anti-VEGF nAMD therapy [16]. In this study, variant rs10490924 (10:124,214,448) achieved a *p*-value of 0.001 in a combined analysis of 22 cohorts (2.917 good responders and 3.600 poor responders). The heterogeneity statistic I^2^ of this test was 86.4%, pointing towards a substantial heterogeneity between studies [16]. This observation illustrates that study-specific effects due to heterogeneity or random sampling errors have a large impact on the results, also for directed approaches.

The origin of study-specific effects could be related to patient phenotyping and the definition of treatment response. For example, in our observational study, the criterion defining a dry retina as a positive treatment outcome classified 107 (59.8%) of 179 patients as responders. Several studies suggest the inclusion of additional markers such as CRT or VA [36]. Still, these parameters have considerable limitations. In the case of VA, we observed a substantial variability although the mean VA remained largely consistent regardless of treatment. Similarly, measurements of CRT may vary between individual eyes, especially if the retina has already developed atrophic areas resulting in CRT thinning. A post-hoc analysis of 210 anti-VEGF-treated nAMD patients demonstrated that the presence of SRF is associated with a higher best corrected VA at all timepoints [37]. Taken together, phenotyping of nAMD treatment responses may vary greatly between centers and even within defined parameters, and thus may introduce a widespread heterogeneity into genetic analysis. We also performed an analysis of genetic effects on CRT but found no statistically significant genetic variant. Nevertheless, our results may be valuable for future meta-analyses and thus were uploaded on Zenodo (doi: 10.5281/zenodo.6579716, accessed 23 May 2022).

Additional parameters, possibly defined in extrafoveolar regions, may be considered and could be beneficial to more precisely define treatment response. Here, we show that TRT seems to offer a more significant separation between responders and non-responders, although this trait still may be insufficient for a truly reliable separation of responders and non-responders. Studies suggesting the use of volumetric measurements to precisely investigate treatment response may be warranted [38,39]. 

An alternative explanation for the study-specific effects we observed may be reflected by different subtypes of responders to nAMD treatment response. For example, retinal angiomatous proliferation (RAP) lesions (macular neovascularization type 3) present a distinct phenotype and have been reported to respond well to anti-VEGF treatment [40,41]. It is likely that there are other subgroups of individual treatment responses that have not yet been characterized. This may be due to subtle phenotypic differences or the fact that many studies are greatly underpowered to define infrequent subtypes. If diverse genetic effects underlie a defined response pattern, it is plausible that different studies report inconsistent results as each cohort likely includes varying subsets of responder phenotypes. In consequence, to avoid inconsistencies in genetic studies which analyze treatment responses, a more refined characterization of treatment phenotypes is needed. In this context, the use of artificial intelligence may prove helpful in identifying subtle response patterns [39]. Subsequently, genetic markers could be developed in the future to assist in characterizing or predicting the different response patterns. Of note, it is also possible that only weak or no genetic effects underlie anti-VEGF treatment response in nAMD. In any case, much larger samples are needed to provide a definite answer to this question. At present, the many conflicting results hinder translation of genetic data into clinical management.

## 5. Conclusions

Our findings highlight that current clinical parameters used to determine treatment success are insufficient to apply meaningful genetic association studies. For example, in all patients treated, the thickness around the fovea decreased significantly in responders as well as in non-responders. Further, the thirty genetic variants reported so far to be significantly associated with treatment response in at least one study failed to be replicated in others, as well as in our cohort. We conclude that the phenotype “anti-VEGF treatment response” is insufficiently defined and requires a more precise characterization before further genetic analyses can be applied successfully. We suggest that future GWAS studies should include patients defined by resilient outcome parameters and a study size of at least 15,000 individuals.

## Figures and Tables

**Table 1:** Study characteristics.

	All	Responder	Non-Responder
*n*	179	128	51
Mean age (SD)	77.3 (7.15)	77.98 (6.56)	75.57 (8.27)
Male/Female	72/107	48/80	24/27
Eylea/Lucentis	63/116	50/78	13/38
Mean VA BSL [logMAR] (SD)	0.53 (0.34)	0.56 (0.36)	0.45 (0.28)
Mean VA treated [logMAR] (SD)	0.44 (0.33)	0.44 (0.33)	0.44 (0.32)
Mean CRT BSL [µm] (SD)	396.47 (158.61)	411.2 (175.4)	359.51 (97.29)
Mean CRT treated [µm] (SD)	266.15 (95.43)	243.74 (80.02)	322.37 (107.92)
Mean TRT BSL [µm] (SD)	500.26 (135.91)	515.93 (149.7)	460.92 (81.45)
Mean TRT treated [µm] (SD)	341.55 (83.44)	322.3 (73.6)	389.88 (87.73)

BSL: baseline; CRT: central retinal thickness; M3: measurement after three monthly treatments; SD: standard deviation; TRT: thickest retinal thickness; VA: visual acuity [logMAR].

**Table 2:** Replication of genetic variants previously associated with anti-VEGF treatment response.

Variant	Locus	Position [hg19]	Effect Allele	Other Allele	Independent Validation Corrected *p*-Value ^a^
rs10158937	*OR52B4*	1:66144876	C	A	0.987
rs12138564	*CCT3*	1:156291600	T	G	0.987
rs3753394	*CFH*	1:196620917	C	T	0.987
rs800292	*CFH*	1:196642233	A	G	0.645
rs1061170	*CFH*	1:196659237	T	C	0.987
rs1329428	*CFH*	1:196702810	T	C	0.987
rs1065489	*CFH*	1:196709774	G	T	0.987
rs17793056	*CX3CR1*	3:39309215	C	T	0.645
rs6828477	*VEGFR2*	4:55966801	T	C	0.987
rs4576072	*VEGFR2*	4:55986238	T	C	0.987
rs2071559	*VEGFR2*	4:55992366	G	A	0.987
rs4073	*IL-8*	4:74606024	T	A	0.987
rs429608	*C2*	6:31930462	A	G	0.987
rs699946	*VEGFA*	6:43732669	G	A	0.645
rs699947	*VEGFA*	6:43736389	C	A	0.987
rs3025000	*VEGFA*	6:43746169	T	C	0.987
rs3025039	*VEGFA*	6:43752536	T	C	0.987
rs2069845	*IL6*	7:22770149	G	A	0.645
rs1883025	*ABCA1*	9:107664301	T	C	0.645
rs25681	*C5*	9:123780005	A	G	0.987
rs2070296	*NRP1*	10:33552695	C	T	0.645
rs10490924	*ARMS2*	10:124214448	G	T	0.987
rs4910623	*OR52B4*	11:4389639	A	G	0.645
rs12366035	*VEGFB*	11:64004692	T	C	0.987
rs55732851	*VWA3A*	16:22137603	G	A	0.645
rs1800775	*CETP*	16:56995236	C	A	0.645
rs12603486	*SERPINF1*	17:1667724	G	A	0.645
rs13900	*CCL2*	17:32583911	T	C	0.987
rs323085	*OR52B4*	18:49290621	G	A	0.987
rs7412	*APOE*	19:45412079	C	T	0.987

^a^ Corrected *p*-value (false discovery rate) of the association model based on our observational study (*n* = 179).

## Data Availability

GWAS summary statistics will be uploaded to the Zenodo online database after the article has been accepted. All further data generated or analyzed during this study are included in this published article and its supplementary information files.

## Supplementary Materials

The following are available online at https://www.mdpi.com/article/10.3390/ijms23116094/s1.
