# Supplementary material for: Genetic Association Analysis of Anti-VEGF Treatment Response in Neovascular Age-Related Macular Degeneration
Source: Int J Mol Sci. 2022 May 29;23(11):6094. doi: 10.3390/ijms23116094 (PMC9181567; doi:10.3390/ijms23116094)
Supplement: Supplementary file 1 [file ijms-23-06094-s001.zip › ijms-1735715-supplementary/Supplementary information file.pdf]

Genetic association analysis for anti-VEGF treatment in neovascular age-related macular degeneration

Tobias Strunz , Michael Pöllmann, Maria-Andreea Gamulescu, Svenja Tamm and Bernhard H. F. Weber

Supplementary Information Files containing

- **Supplementary Figures S1 to S3 and**
- **Supplementary Tables S1 and S2**

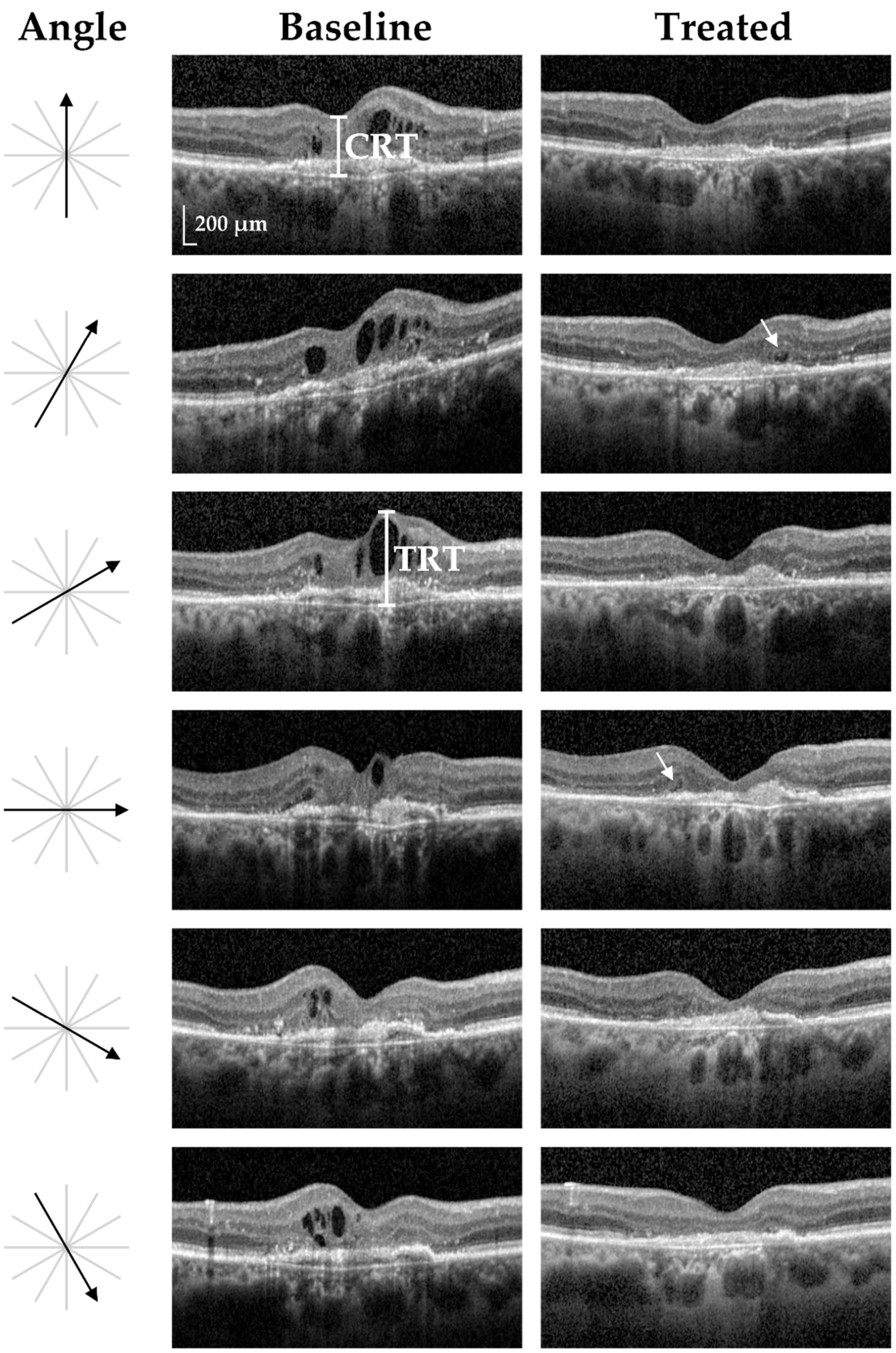

Supplementary Figure S1. Exemplary case report. Shown are OCT images from an 80-year-old female patient before and after treatment. The patient was defined as non-responder as some intraretinal fluid remained after treatment (arrows). Central retinal thickness (CRT) was determined measuring the spacing between Bruch's Membrane and the Internal Limiting Membrane at the fovea centralis. Analysis of retinal thickness revealed vast differences depending on the angle. Thickest retinal thickness (TRT) was identified by manual screening of six-line scans from different angles.

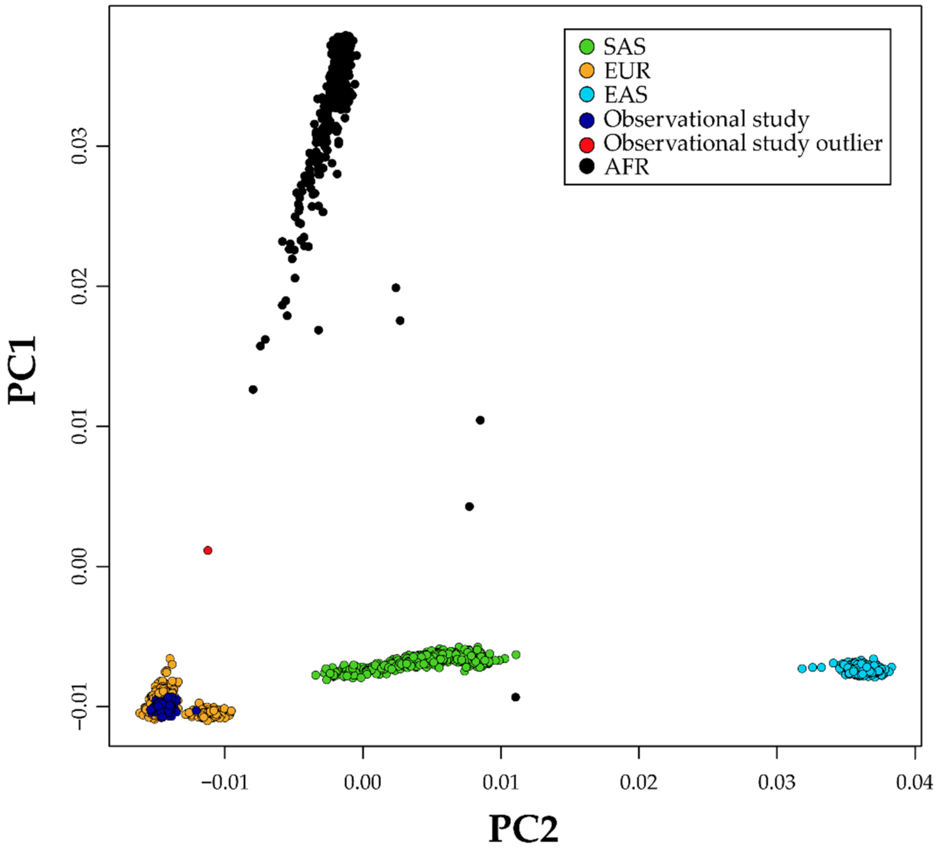

Supplementary Figure S2. Genotype principal component analysis (PCA). 100,000 autosomal variants were randomly extracted from 180 individuals of the observational study. In addition, the genotypes from samples of European (EUR, yellow), African (AFR, black), South Asian (SAS, green), and East Asian (EAS, light blue) ancestry were selected from the 1000 Genomes Project [28]. PCA was performed and the first two principal components (PC1 and PC2) of the 180 samples from our observational study (dark blue) were plotted. One sample (red) did not cluster next to the EUR reference and was excluded from further association analysis.

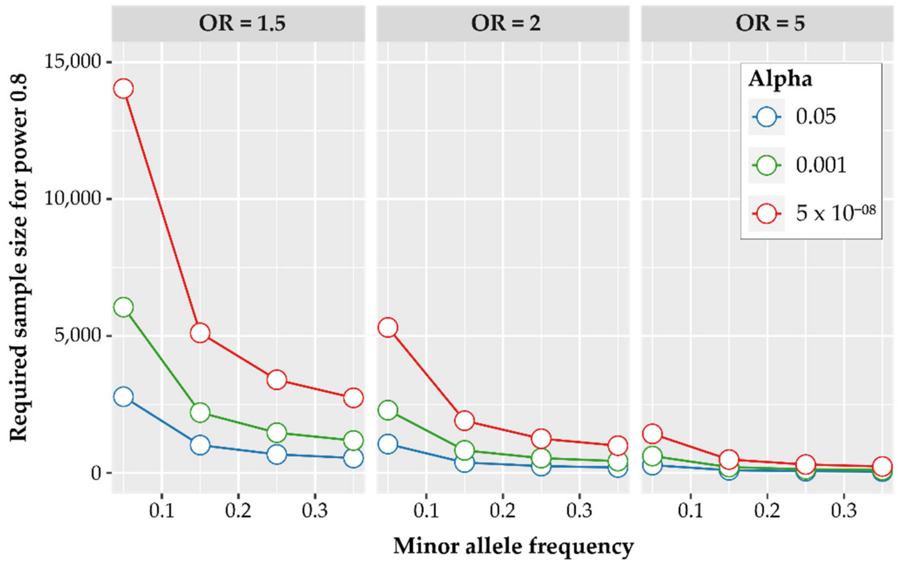

Supplementary Figure S3. Sample size requirements to achieve a statistical power of 0.8. Shown are the required sample sizes (y-axis) to achieve a power of 0.8 under varying conditions. Power analysis was performed for a case-control study based on a case rate of 71.5 % as measured in our observational study. Different alpha levels represent three scenarios: single variant testing (alpha 0.05, blue), multiple variant testing adjusted for multiple testing (alpha 0.001, green), and genome-wide testing (alpha 5×10^{-08}, red). The Odds ratio (OR) of the underlying genetic effect is depicted in three panels, which are further divided regarding the minor allele frequency of the genetic variant (x-axis).

Supplementary Tables (see separate Excel spead sheet)

Supplementary Table S1. Studies investigating the genetics of anti-VEGF nAMD treatment from 2014 to 2021

Supplementary Table S2. Variants suggested to be associated with anti-VEGF nAMD treatment response in previous publications.
